# A Mathematical Model for Determining Probabilistic Design Space in Mesenchymal Stem Cell Passage Culture

**DOI:** 10.1002/bit.29001

**Published:** 2025-04-25

**Authors:** Keita Hirono, Yusuke Hayashi, Yuuki Ogawa, Masahiro Kino‐oka, Hirokazu Sugiyama

**Affiliations:** ^1^ Department of Chemical System Engineering The University of Tokyo Tokyo Japan; ^2^ Department of Biotechnology, Graduate School of Engineering The University of Osaka Osaka Japan

**Keywords:** design space, mathematical modeling, quality by design (QbD), regenerative medicine, stem cell manufacturing, stochastic simulation

## Abstract

With their many therapeutic functions, mesenchymal stem cells (MSCs) are promising sources for regenerative medicine. However, in the manufacture of MSCs, without a method for exploring the effects of long‐term passage on cell proliferation potentials, the design of passage culture processes is challenging. Here, for the process design of the MSC passage culture, we propose a model for predicting the growth rate as a function of the cumulative population doubling level (cPDL) for each passage. Three steps were implemented: (1) passage culture experiments to correlate apparent growth rate with cPDL were conducted, (2) a model for predicting the growth rate as a function of cPDL was developed, and (3) a model to design the passage culture of MSCs from bone marrow (BM‐MSCs) and umbilical cord (UC‐MSCs) with stochastic simulation was applied. Two design variables (passage number and harvesting time) were investigated to define feasible operation regions as probabilistic design spaces to meet three quality indicators (senescence level, confluency level, and total number of cells) with given probabilities. Consequently, 10 and 62 conditions out of 165 were identified as feasible for BM‐ and UC‐MSCs, respectively, which would contribute to the industrial MSC passage culture process design.

## Graphical abstract

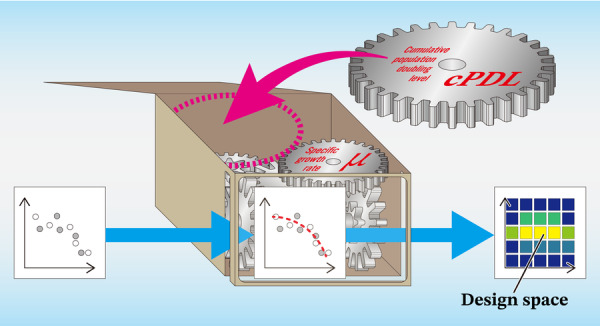

**Abbreviations**
BM: bone marrow
cPDL: cumulative population doubling level
DS: design space
MSC: mesenchymal stem cell
NRMSE: normalized root mean square error
PDL: population doubling level
QI: quality indicator
RSS: residual sum of squares
UC: umbilical cord

## 1 Introduction

Human mesenchymal stem cells (MSCs) are promising sources for regenerative medicine with their many therapeutic functions, including multilineage potentials (Pittenger et al. 1999). There are increased demands for therapeutic MSCs with a wide range of clinical targets such as cardiovascular diseases and immunological disorders (Heathman et al. 2015). To address the anticipated demand for MSCs, a sequential passage culture is a crucial prior step to clinical use (Li et al. 2012). In passage culture processes, single cultivation is repeated to yield a planned number of cells where the cultivation conditions and the passage number are key parameters. Passage culture is susceptible to cell‐source variability such as isolation sources (e.g., bone marrow, umbilical cord, adipose tissue) (Kern et al. 2006) and donors (e.g., age (Zaim et al. 2012), and gender (Fossett et al. 2012)).

There are quality concerns due to the replicative senescence of MSCs during passage culture processes (Digirolamo et al. 1999; Mets and Verdonk 1981). It is the case with MSCs that human diploid cells have limits to their growth potential (Hayflick 1965); specifically, the growth rate decreases as the cumulative population doubling level (cPDL) increases during passage culture (Bruder et al. 1997). For example, the growth rate of MSCs from bone marrow (BM‐MSCs) and umbilical cord (UC‐MSCs) decelerated when their cPDL approached ~30 (Bonab et al. 2006) and ~50 (Wiese et al. 2019), respectively. Furthermore, the increase in cPDL by long‐term passage culture impairs the immunosuppressive effects of MSCs (Li et al. 2012). Senescence was experimentally investigated with a focus on cPDL, but the effects of cPDL on long‐term passage for cell proliferation potentials were not fully understood, which made it difficult to design passage culture considering senescence.

A mathematical model that enhances systematic understanding by connecting critical quality attributes (CQAs) with critical process parameters (CPPs) complying with a Quality by Design framework (ICH 2009) could be a strong tool to overcome this difficulty. Models play a key role in defining design spaces (DSs) with a comparatively small number of experiments (Destro and Barolo 2022). By exploring every combination of CPPs, feasible operation regions can be identified as DSs such that all CQAs can meet quality specifications. In MSC cultivation, for example, DSs were determined as feasible operation regions of seeding density and medium change ratio (i.e., CPPs) such that the confluency and total number of cells (i.e., CQAs) predicted by a kinetic model could satisfy quality specifications (Hirono et al. 2022). To date, a few models have been developed to predict the growth rate for a given passage culture condition. A stochastic model was implemented, in which two different degrees of damage were applied to individual cells in each passage considering the initial population heterogeneity (Jin et al. 2017), whereas a machine learning model directly correlated population doubling time and donor characteristics (Mehrian et al. 2020). However, from a process design perspective, further model development is needed to consider the senescence effects for a wide range of passage culture conditions.

In this study, we aimed to develop a model to predict growth rate as a function of cPDL for each passage for the MSC passage culture process design. Three steps were implemented: (1) passage culture experiments to correlate the growth rate with cPDL were conducted, (2) a model was developed to predict the growth rate as a function of cPDL, and (3) a model with stochastic simulation for the design of passage culture of BM‐MSCs and UC‐MSCs was applied. Consequently, feasible operation regions of the passage number and harvesting time were defined as probabilistic DSs such that senescence and confluency levels and the total number of cells could meet quality specifications with given probabilities. The developed model could be extended to other design variables (e.g., seeding density) and integrated with unit operation models (e.g., seeding, cryopreservation) toward the process design of industrial MSC passage culture.

## 2 Materials and Methods

### 2.1 Passage Culture Experiment

**Figure 1:**
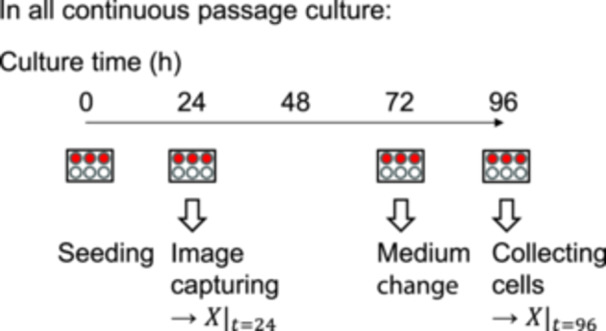
Schematic representation of the mesenchymal stem passage culture process. Triplicate samples (in red) were investigated independently through the passage culture, whereas the remaining wells were not used (in white).

In the following experiments, BM‐derived MSCs (Lot no. 19TL337479; Lonza, Walkersville, MD, USA) and UC‐derived MSCs (provided by the Hyogo College of Medicine, Hyogo, Japan) were used. For all experiments, MSCs were harvested in MSCGM (Lonza) at 37°C in a humidified atmosphere containing 5% CO_2_. The seeding density was 5.0 × 10^3^ cells cm^–2^, and medium changes were performed every 3 days. At 1‐day cultivation, phase contrast images were captured using an imaging system (EVOS XL Core; Thermofisher Scientific, Waltham, MA, USA) at four points, randomly. After 4‐day cultivation, the cells were passaged by enzymatic treatment with a trypsin/ethylenediaminetetraacetic acid solution (Thermofisher Scientific) with counting cells by an auto‐cell counter (TC‐20; Bio‐rad, Hercules, CA, USA). The harvested cells were then seeded for the next single cultivation with the same seeding density (i.e., 5.0 × 10^3^ cells cm^–2^) under fresh culture medium conditions (Figure 1).

The specific growth rate in the exponential growth phase, composed of the collected value of cell density and the total value of the attached cell density counted from the captured images, is given as follows:

(1) $$\mu^{\text{exp}}=\frac{\ln\left(\frac{{\mathrm{X|}}_{t=96}}{{\mathrm{X|}}_{t=24}}\right)}{96-24},$$

where $\mu^{\text{exp}}$ is the specific growth rate obtained from the experimental data, ${\mathrm{X|}}_{t=24}$is the density of cells counted from the images taken 24 h after seeding, and ${\mathrm{X|}}_{t=96}$ is the density of cells collected 96 h after seeding.

### 2.2 Model Development for Passage Culture Processes

**Figure 2:**
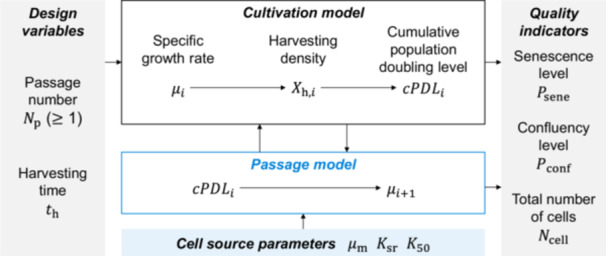
Parameter map of the developed model for the passage culture processes. The parameter $\mathrm{cPD}L_{i}$ evolves along the iterative process (i= 1, 2, …, $N_{p}$), while $\mu_{m}$, $K_{\mathrm{sr}}$, and $K_{50}$ remain constant throughout the passage culture process.

To represent the passage culture processes, a passage model was developed and integrated with a typical cultivation model, connecting three sets of parameters, the design variables, cell‐source parameters, and quality indicators (QIs) (Figure 2). In particular, the design variables and the cell‐source parameters were used as inputs to the cultivation and passage models, respectively, to predict the QIs; the passage model was executed to predict the specific growth rate, μ, that could be used as inputs for the next cultivation. Here, two design variables of passage culture were defined, namely, the passage number, $N_{p}$, which determines the number of iterations of a single cultivation, and the harvesting time, $t_{h}$, which determines the cultivation time for a single cultivation. Together, three QIs were defined after each passage, as follows: the senescence level, $P_{\text{sene}}$, the confluency level, $P_{\text{conf}}$, and the total number of cells, $N_{\text{cell}}$. Finally, the cultivation and passage models were used to identify the feasible operation regions of $N_{p}$ and $t_{h}$, such that all the predicted values, $P_{\text{sene}}$, $P_{\text{conf}}$, and $N_{\text{cell}}$ satisfied the quality specifications.

In the cultivation model, typical exponential growth and stationary phases were used. Specifically, during the growth phase, μ was assumed to be constant, whereas the contact inhibition due to confluency was applied to represent the stationary phase. The harvesting density, $X_{h,i}$, after the i‐th single cultivation was predicted as follows:

(2) $$X_{h,i}=\min\left\{X_{s}\text{exp}\left(\mu_{i}t_{h}\right),X_{m}\right\},$$

where $\mu_{i}$ is the specific growth rate during the i‐th single cultivation (constant during single cultivation), $X_{m}$ is the maximum cell density, $X_{s}$ is the seeding density, and min{∙} is the function that takes the minimum value from the inputs. Here, $X_{m}$ is the largest value of the experimental measurements (including previously reported values) (Hirono et al. 2024) and remains constant throughout the passage culture. Following the end of the i‐th single cultivation, the resulting $X_{h,i}$ was used to calculate cPDL as follows:

(3) $$\mathrm{cPD}L_{i}=\sum_{j=1}^{i}\mathrm{PD}L_{j}=\sum_{j=1}^{i}\log_{2}\left(\frac{X_{h,j}}{X_{s}}\right),$$

where $\mathrm{cPD}L_{i}$ is the calculated cPDL resulting from the i‐steps of single cultivation (to be inherited by the (i+1)‐th cultivation) and $\mathrm{PD}L_{i}$ is the calculated population doubling level (PDL) resulting from the i‐th single cultivation (to be recalculated for the (i+1)‐th cultivation). It was assumed that cPDL remained constant as $\mathrm{cPD}L_{i-1}$ during the i‐th single cultivation and was then updated to $\mathrm{cPD}L_{i}$ by Equation (3) before the (i+1)‐th cultivation. Finally, the resulting $\mathrm{cPD}L_{i}$ was used as input for the following passage model, which allowed $\mu_{i+1}$ prediction by accounting for the effects of senescence.

(4) $$\mu_{i+1}=\mu_{m}\frac{1}{1+\text{exp}\left\{K_{\text{sr}}\left(\mathrm{cPD}L_{i}-K_{50}\right)\right\}},$$

where $\mu_{m}$ is the maximum specific growth rate, $K_{\text{sr}}$ is the senescence rate, and $K_{50}$ is the passage model constant representing cPDL when μ decreases by 50% from $\mu_{m}$. The three parameters (i.e., $\mu_{m}$, $K_{\text{sr}}$, $K_{50}$) were estimated using the experimental data to incorporate cell‐source variability and remained constant throughout the passage culture. Finally, the resulting $\mu_{i+1}$ was used for the next cultivation, i.e., the calculation proceeded and returned to Equation (2).

Together with the iterative process consisting of Equations (2)–(4), the number of flasks to be used for the next cultivation was also calculated at the end of the i‐th cultivation as follows:

(5) $$n_{\text{flask},i+1}=\min\left(\left\lfloor \frac{n_{\text{flask},i}X_{h,i}}{X_{s}}\right\rfloor ,n_{\text{flask}}^{\max}\right),$$

where $n_{\text{flask},i}$ is the number of working flasks for the i‐th cultivation, $n_{\text{flask}}^{\max}$ is the maximum number of available flasks (constant throughout passage culture), and ⌊∙⌋ is the floor function that takes the greatest integer less than or equal to the input.

After the end of the passage culture, the models' outputs were used to predict the QIs. Specifically, the calculated $X_{h,N_{p}}$, $\mu_{N_{p}+1}$, and $n_{\text{flask},N_{p}}$ were used for the predictions of $P_{\text{sene}}$, $P_{\text{conf}}$, and $N_{\text{cell}}$ as follows:

(6) $$P_{\text{sene}}=\frac{\mu_{N_{p}+1}}{{\mu |}_{\mathrm{cPDL}=0}},$$

(7) $$P_{\text{conf}}=\frac{X_{h,N_{p}}}{X_{m}},$$

(8) $$N_{\text{cell}}=n_{\text{flask},N_{p}}SX_{h,N_{p}},$$

where ${\mu |}_{\mathrm{cPDL}=0}$ is the initial value of μ when cPDL is numerically 0 (see also Equation (4)), and S is the surface area of the flask. Here, we defined $P_{\text{sene}}$ to represent quantitatively how far the resulting μ decreased from the value before starting the passage culture. From the definition, a larger $P_{\text{sene}}$ is less affected by senescence, and $P_{\text{sene}}$ decreases as the passage culture progresses. Regarding the confluency, we applied a previous indicator to estimate confluency (Hirono et al. 2024); in particular, a smaller $P_{\text{conf}}$ is better, and this parameter increases as growth progresses during a single cultivation. Finally, the total number of cells to be harvested was defined with the number of working flasks considering the maximum number of flasks.

### 2.3 Parameter Estimation and Model Validation

Parameter estimation and model validation were performed to apply the developed model to the passage culture process design. For each cell source, experimental data from three independent samples were used; two samples (e.g., samples 1 and 2) were used to estimate the three parameters in Equation (4) (i.e., $\mu_{m}$, $K_{\text{sr}}$, and $K_{50}$), and the one remaining sample (e.g., sample 3) was used to validate the model. Only Equation (4) was fitted to the two samples using cPDL as obtained from the experiments, whereas the entire model (i.e., Equations (2)–(4)) was validated against the remaining sample. This procedure was performed three times with different sample combinations to calculate the mean of the three estimations for each parameter. Specifically, these parameters were estimated by minimizing the residual sum of squares (RSS) and were evaluated with the normalized root mean square error (NRMSE) between the experimental data and prediction of μ for the same cPDL as follows:

(9) $$\mathrm{RSS}=\sum_{i}^{n}\sum_{j}^{m}{\left(\mu_{i,j}^{\text{exp}}-\mu_{i,j}\right)}^{2},$$

(10) $$\mathrm{NRMSE}=\frac{\sqrt{\frac{1}{n∙m}\sum_{i}^{n}\sum_{j}^{m}{\left(\mu_{i,j}^{\text{exp}}-\mu_{i,j}\right)}^{2}}}{\max\left(\mu^{\text{exp}}\right)-\min\left(\mu^{\text{exp}}\right)}\times 100\%,$$

where m is the number of measurements, n is the number of samples, and $\max\left(\mu^{\text{exp}}\right)$ and $\min\left(\mu^{\text{exp}}\right)$ are the maximum and minimum values of $\mu^{\text{exp}}$, respectively. Subsequently, the NRMSE between the prediction and the remaining μ data for the same passage number was calculated for the model validation.

### 2.4 Stochastic Simulation of Specific Growth Rate and QI

The stochastic simulation was performed based on the experimental data, considering variations inherent in the passage culture processes. First, to quantify the resulting variations in $\mu^{\text{exp}}$, a random variable, e, was defined with the experimental data of the three independent samples. Here, the sample standard deviation was calculated between the three measurements of $\mu^{\text{exp}}$ at each passage number to assume a normal distribution to represent likely variations in μ; the definitions are given as follows:

(11) $$\bar{\mathrm{SD}}=\frac{1}{N_{p}^{\text{exp}}}\sum_{i}^{N_{p}^{\text{exp}}}\sqrt{\frac{1}{n-1}\sum_{j}^{n}{\left(\mu_{i,j}^{\text{exp}}-\bar{\mu_{i}^{\text{exp}}}\right)}^{2}},$$

(12) $$e\sim N\left(0,\bar{\mathrm{SD}}\right),$$

where $\bar{\mu^{\text{exp}}}$ is the mean of $\mu^{\text{exp}}$, e is the random variable to represent the variability, n is the number of samples, N(a,b) is the normal distribution with the mean a and the standard deviation, b, $N_{p}^{\text{exp}}$ is the passage number in the experiment, and $\bar{\mathrm{SD}}$ is the mean of the standard deviation.

Second, the random variable, e, was incorporated into the passage model to simulate likely variations in μ as follows:

(13) $$\mu_{i+1}=\mu_{m}\frac{1}{1+\text{exp}\left\{K_{\text{sr}}\left(\mathrm{cPD}L_{i}-K_{50}\right)\right\}}+e.$$

For the stochastic simulation, e was randomly sampled from the distribution, which generated the variation in $\mu_{i+1}$ for the subsequent predictions. The $\mu_{i+1}$ was then used as an input of the next cultivation model (Equation (2)) followed by the passage model, which was repeated until the end of the passage culture.

Third, the cultivation and passage models were applied to simulate likely variations in $P_{\text{sene}}$, $P_{\text{conf}}$, and $N_{\text{cell}}$ at the end of the passage culture. The simulated $X_{h,N_{p}}$ was used to predict $P_{\text{conf}}$ (Equation (7)) and $N_{\text{cell}}$ (Equation (8)), whereas the $\mathrm{cPD}L_{N_{p}}$ was applied to predict $\mu_{N_{p}+1}$ with the passage model (Equation (13)). The resulting $\mu_{N_{p}+1}$ value was used for the predictions of $P_{\text{sene}}$ (Equation (6)). Here, $P_{\text{sene}}$ could exceed 1 when the calculated $\mu_{N_{p}+1}$ was larger than the ${\mu |}_{\mathrm{cPDL}=0}$, which was constant. This simulation was iterated 10000 times for a given passage culture condition (i.e., $N_{p}$ and $t_{h}$).

### 2.5 Design Space Determination

The stochastic simulation results were applied to illustrate feasible sets of the design variables such that all the predicted QIs ensured the following quality specifications:

(14) $$\begin{array}{c} A=\{\left(P_{\text{sene}},P_{\text{conf}},N_{\text{cell}}\right)|0.8\;\leq P_{\text{sene}}\wedge P_{\text{conf}}<0.9\wedge 3.5\;\times 10^{8}\leq N_{\text{cell}}\},\\ A_{1}=\{P_{\text{sene}}|0.8\leq P_{\text{sene}}\},\\ A_{2}=\left\{P_{\text{conf}}|P_{\text{conf}}<0.9\right\},\\ A_{3}=\left\{N_{\text{cell}}|3.5\times 10^{8}\leq N_{\text{cell}}\right\}, \end{array}$$

where A is the set of the quality specifications of the senescence level, $A_{1}$; the confluency level, $A_{2}$; and the total number of cells, $A_{3}$. The threshold of $P_{\text{sene}}$ was defined in this study as 80% such that the senescence could not proceed. The threshold of $P_{\text{conf}}$ was specified based on prior works where MSCs were passaged before confluency reached 90% (Bruder et al. 1997; Zaim et al. 2012), and that of $N_{\text{cell}}$ was determined based on the previously described dose for myocardial infarction (Lipsitz et al. 2016).

For every combination of the design variables (i.e., $N_{p}$ and $t_{h}$), the probability of ensuring the specifications was calculated with a prior algorithm (Hirono et al. 2024) to define a probabilistic DS. By comparing the probability and a user‐specified minimum acceptable risk, π (García‐Muñoz et al. 2015), the feasible operation regions of $N_{p}$ and $t_{h}$ were specified such that the probability was equal to or greater than π. Here, 165 design variable combinations were numerically explored based on the experimental conditions; in particular, $N_{p}$ was set from 1 to 15 (a total of 15 conditions), whereas $t_{h}$ was investigated from Day 2.5 to 5.0 every 0.25 day (a total of 11 conditions).

(15) $$\begin{array}{c} \mathrm{DS}=\{\left(N_{p},t_{h}\right)\in L^{n}|h\left(N_{p},t_{h}\right)\geq \;\pi \},\\ s.t.\\ h\left(N_{p},t_{h}\right)=\frac{1}{M}\sum_{i=1}^{M}I\left(y_{i}\in A\right)\times 100\%,\\ y=f\left(N_{p},t_{h},e\right), \end{array}$$

where f is the developed model, h is the calculated probability, I(∙) is an indicator function, which takes a value of 1 if all conditions are satisfied and 0 if at least one condition is not satisfied, $L^{n}$ is every combination of $N_{p}$ and $t_{h}$ (165 conditions in total), M is the number of model iterations for a given $N_{p}$ and $t_{h}$ (i.e., 10000 times), and y is the model prediction of the QIs (i.e., $P_{\text{sene}}$, $P_{\text{conf}}$, and $N_{\text{cell}}$).

The model equations and the algorithm for the stochastic simulation and DS determination were implemented with Python 3.9.13. The total CPU time for the results in Figures 5, 6, 7, 8 was approximately 30 min using an Intel Xeon Gold 6142 CPU @ 2.60 GHz with 128 GB of RAM.

## 3 Results

### 3.1 Cell Growth Through Passage Culture

**Figure 3:**
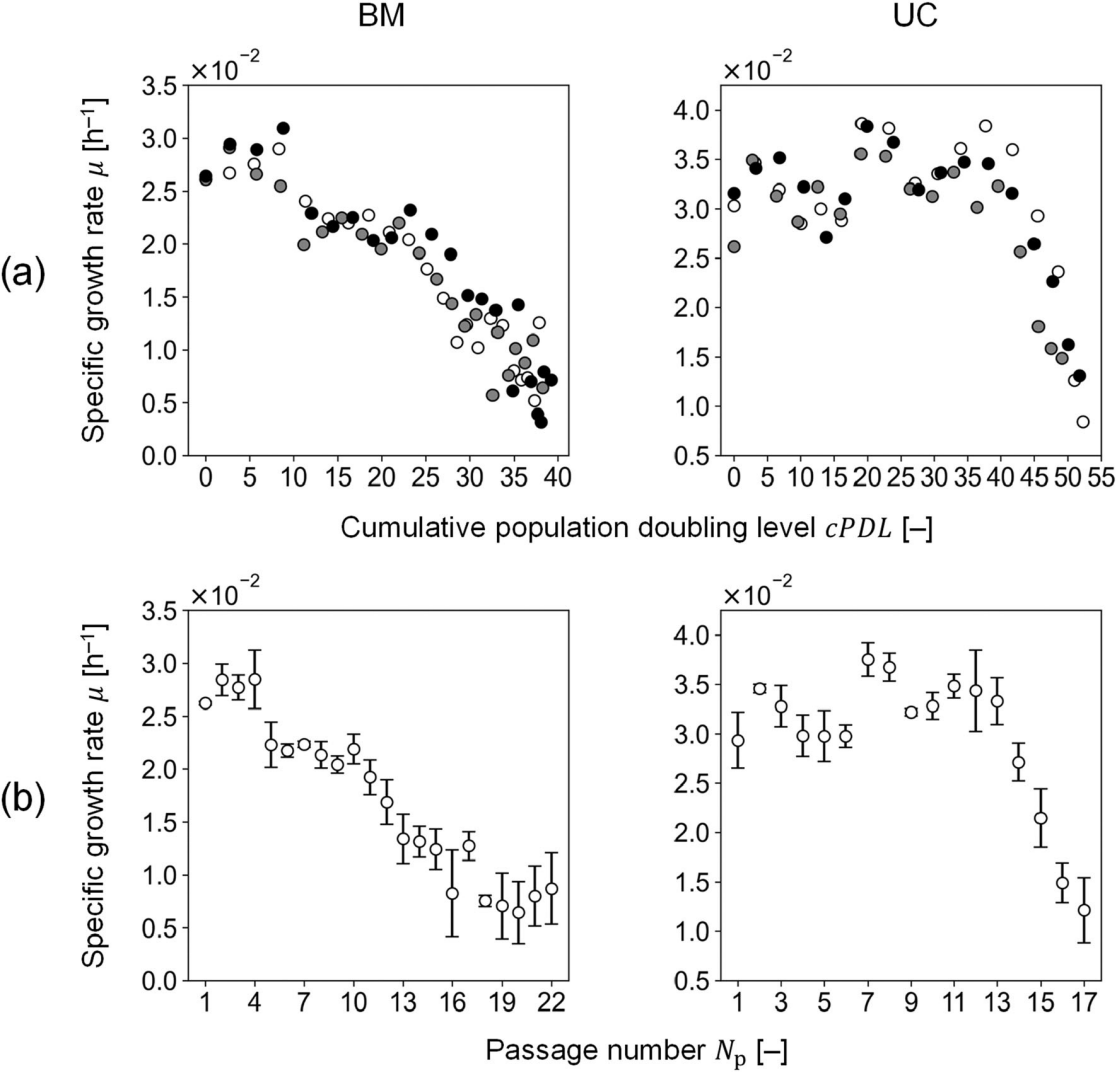
Passage culture results with the harvesting time of Day 4 at every passage. (a) The specific growth rate with respect to the cumulative PDL; color code: white (sample 1), gray (sample 2), and black (sample 3). (b) The specific growth rate refers to the passage number. The white circles show the mean; the error bars represent the standard deviation of the three independent samples. BM and UC represent MSCs from bone marrow and umbilical cord, respectively.

The passage culture experiments yielded changes in μ with respect to cPDL (Figure 3a) and $N_{p}$ (Figure 3b) for both BM‐ and UC‐MSCs. Senescence was observed in both MSCs; specifically, μ of BM‐ and UC‐MSCs started decreasing around the cPDL values of 10 and 40, respectively. BM‐MSCs experienced senescence at the early passage ($N_{p}=$ 5) but the decreasing rate of μ is slow (Figure 3b). By contrast, μ of UC‐MSCs rapidly decreased although the senescence started at a late passage ($N_{p}=$ 13) (Figure 3b). These results indicated that trends in senescence could be interpreted with a focus on when it started and how fast it progressed.

### 3.2 Parameter Estimation and Model Validation

**Figure 4:**
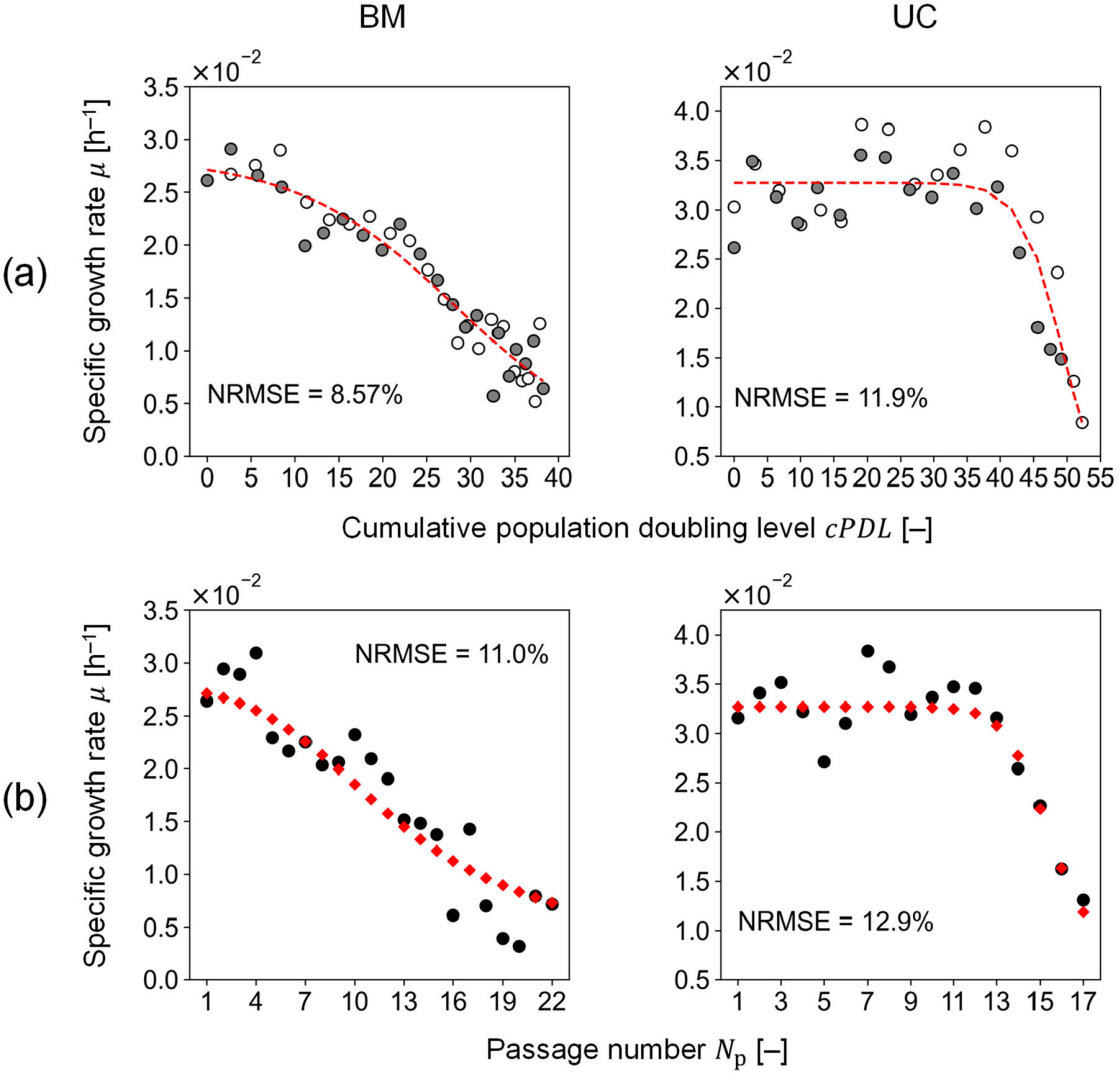
Parameter estimation and model validation results. (a) Parameter estimation with only Equation (4), which was fitted to the two independent samples (i.e., samples 1 and 2) using cPDL as obtained from the experiments; color code: white (sample 1), gray (sample 2), and black (sample 3). Red dashed lines represent the model fit. (b) Model validation with the remaining sample (i.e., sample 3). Red diamond plots show the model prediction. NRMSE is the normalized root mean square error. BM and UC represent MSCs from bone marrow and umbilical cord, respectively.

The parameter estimation of the passage model (i.e., Equation (4)) was individually conducted for BM‐ and UC‐MSCs. The least squares method was applied for each sample set (Supporting Information S1: Table S1), which obtained the mean values of the parameters (Table 1) with resulting NRMSE values smaller than 15% (Figure 4a, and Supporting Information S1: Figure S1). For the model validation, μ was predicted for each passage with the specified value of $X_{m}$ (Supporting Information S1: Table S2), resulting in NRMSE values smaller than 20% (Figure 4b, Supporting Information S1: Figure S1), which indicated that the model could follow the impact of senescence on the growth rate.

**Table 1:** Mean values of estimated parameters. BM and UC represent MSCs from bone marrow and umbilical cord, respectively.

Cell source	Maximum specific growth rate $\mu_{m}$ [10^–2^ h^–1^]	Senescence rate $K_{\text{sr}}$ [–]	Passage model constant $K_{50}$ [–]
BM	2.80	0.120	29.2
UC	3.30	0.332	49.3

### 3.3 Stochastic Simulation of Specific Growth Rate and QIs

With the estimated parameter values of BM‐ and UC‐MSCs, the stochastic simulation was conducted until $N_{p}=$ 15. As a case study, a multilayer flask with a surface area of 1720 cm^2^ was applied for the passage culture simulation where the initial and maximum number of flasks were set as 1 and 10, respectively.

**Figure 5:**
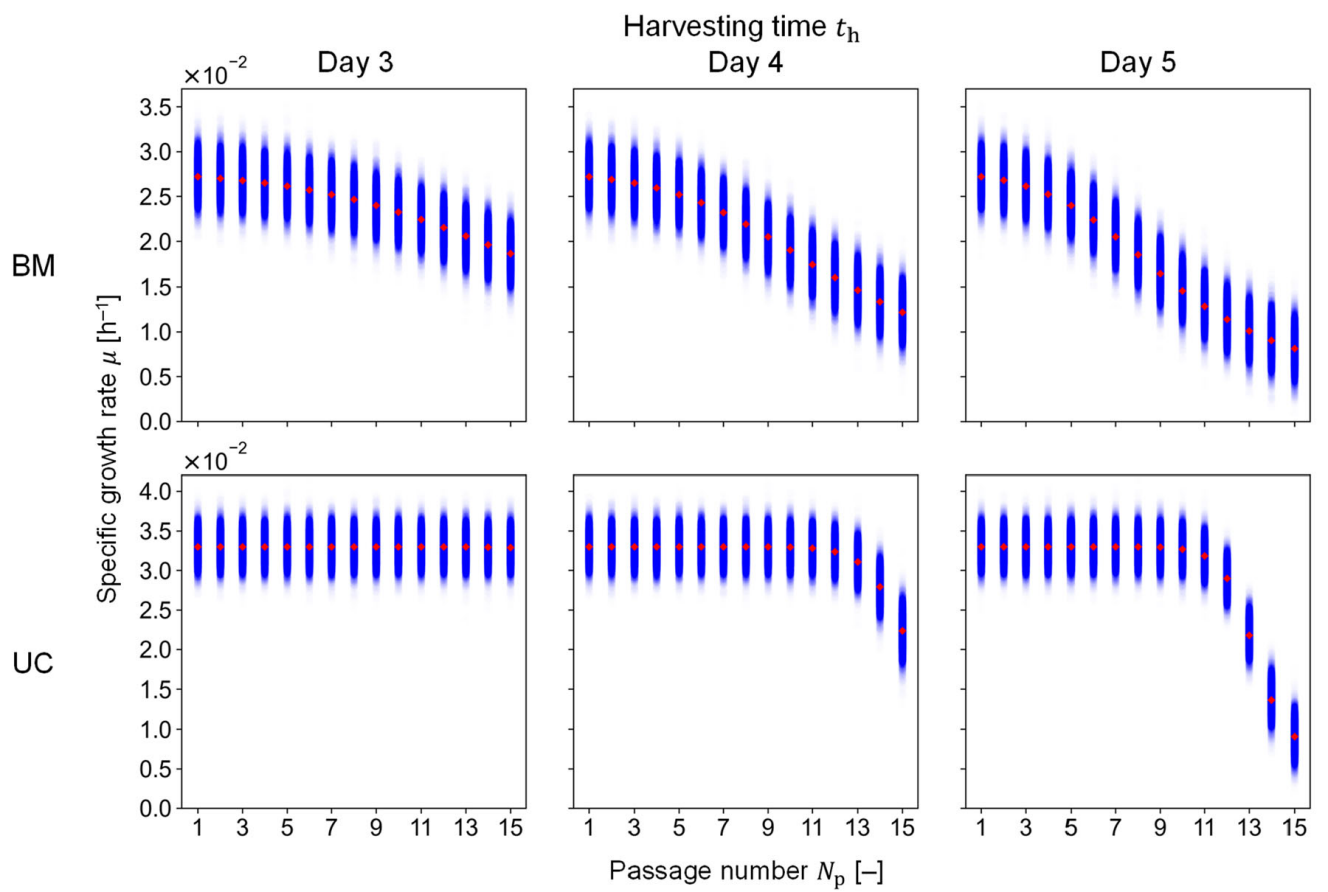
Stochastic simulation results of the specific growth rate. Blue plots show the stochastic simulation results with 10,000 iterations; red diamond plots illustrate the simulation results without stochastic nature; numerically, the random variable value was equal to 0. BM and UC represent MSCs from bone marrow and umbilical cord, respectively.

**Figure 6:**
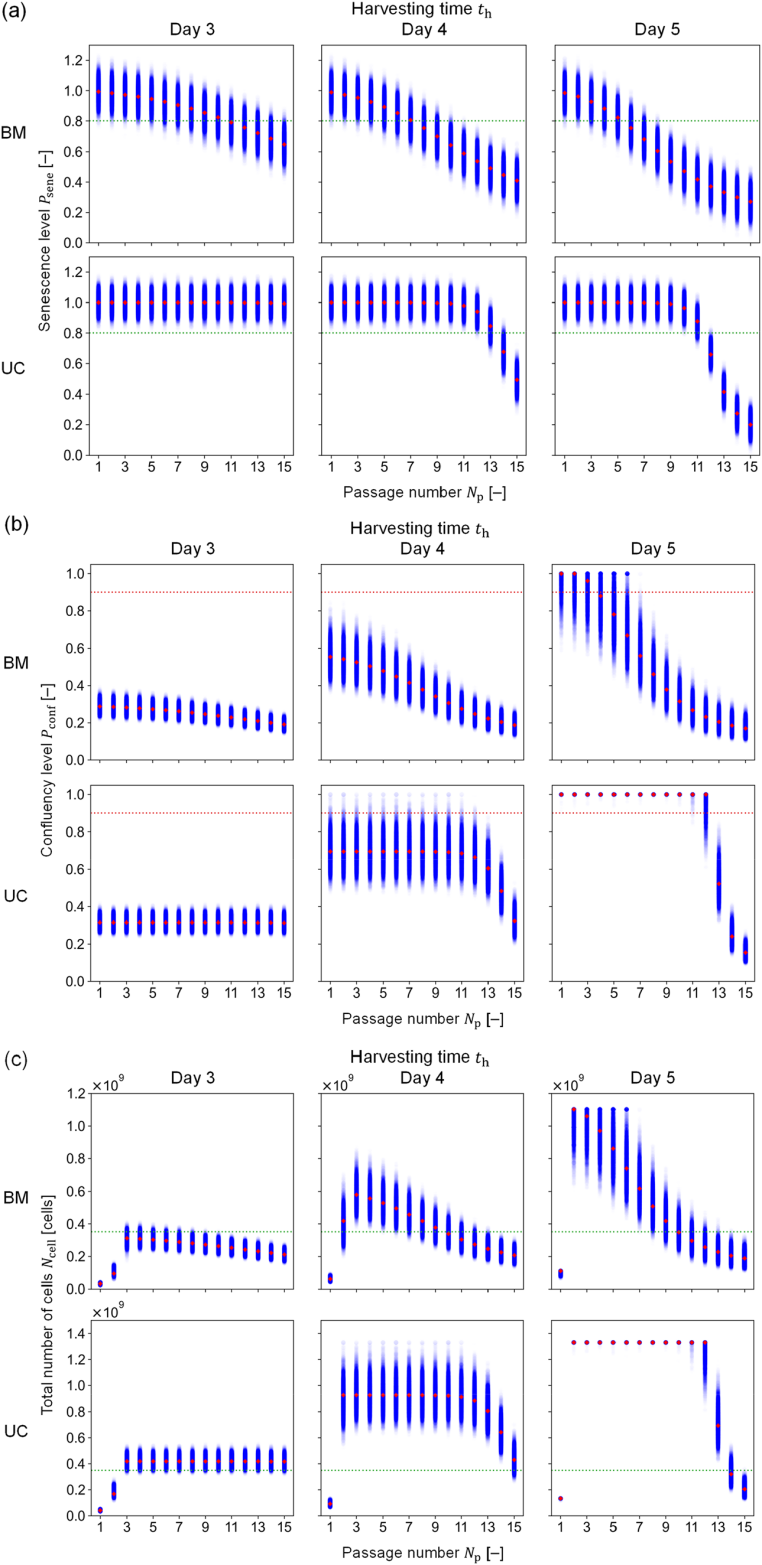
Stochastic simulation results of QIs. Blue plots show the stochastic simulation results with 10,000 iterations; red diamond plots illustrate the simulation results without stochastic nature; numerically, the random variable value was equal to 0. Green and red dotted lines show the lowest and highest acceptable values to meet the quality specification, respectively. (a) Result for the senescence level. (b) Result for the confluency level. (c) Result for the total number of cells. BM and UC represent MSCs from bone marrow and umbilical cord, respectively.

First, the random variable, e, was defined to follow the normal distribution with $\bar{\mathrm{SD}}$ obtained as 1.80 × 10^–3^ h^–1^ and 1.99 × 10^–3^ h^–1^ for BM‐ and UC‐MSCs, respectively. Second, the resulting e was incorporated into the passage model for the stochastic simulation, yielding normally distributed variations in μ for distinct $t_{h}$ values (Days 3–5) (Figure 5). These results indicated that longer $t_{h}$ conditions decreased the growth rate more rapidly; specifically, $t_{h}$ of Day 3 for UC‐MSCs showed no senescence effects on the growth rate. These simulations resulted in the same variation degree, independently of $N_{p}$ and $t_{h}$. Third, the resulting variations in μ were propagated to the QIs of $P_{\text{sene}}$ and $N_{\text{cell}}$ by the cultivation and passage models. The normal distribution nature of μ is reflected into $P_{\text{sene}}$ (Figure 6a) but transformed into the lognormal distribution nature of $P_{\text{conf}}$ (Figure 6b) because of the exponential growth. Specifically, the $P_{\text{sene}}$ resulted in a constant degree of variations (Figure 6a), whereas the variation in $P_{\text{conf}}$ had a lognormal distribution that became narrower as μ decreased (Figure 6b). Moreover, $P_{\text{conf}}$ more frequently reached 1.0 when $t_{h}$ was longer with a lower $N_{p}$ (Figure 6b). Although these trends in $P_{\text{conf}}$ were reflected into $N_{\text{cell}}$, the predicted values of $N_{\text{cell}}$ rapidly increased at the early passage due to the increase in $n_{\text{flask}}$ (Figure 6c).

### 3.4 DS Determination

**Figure 7:**
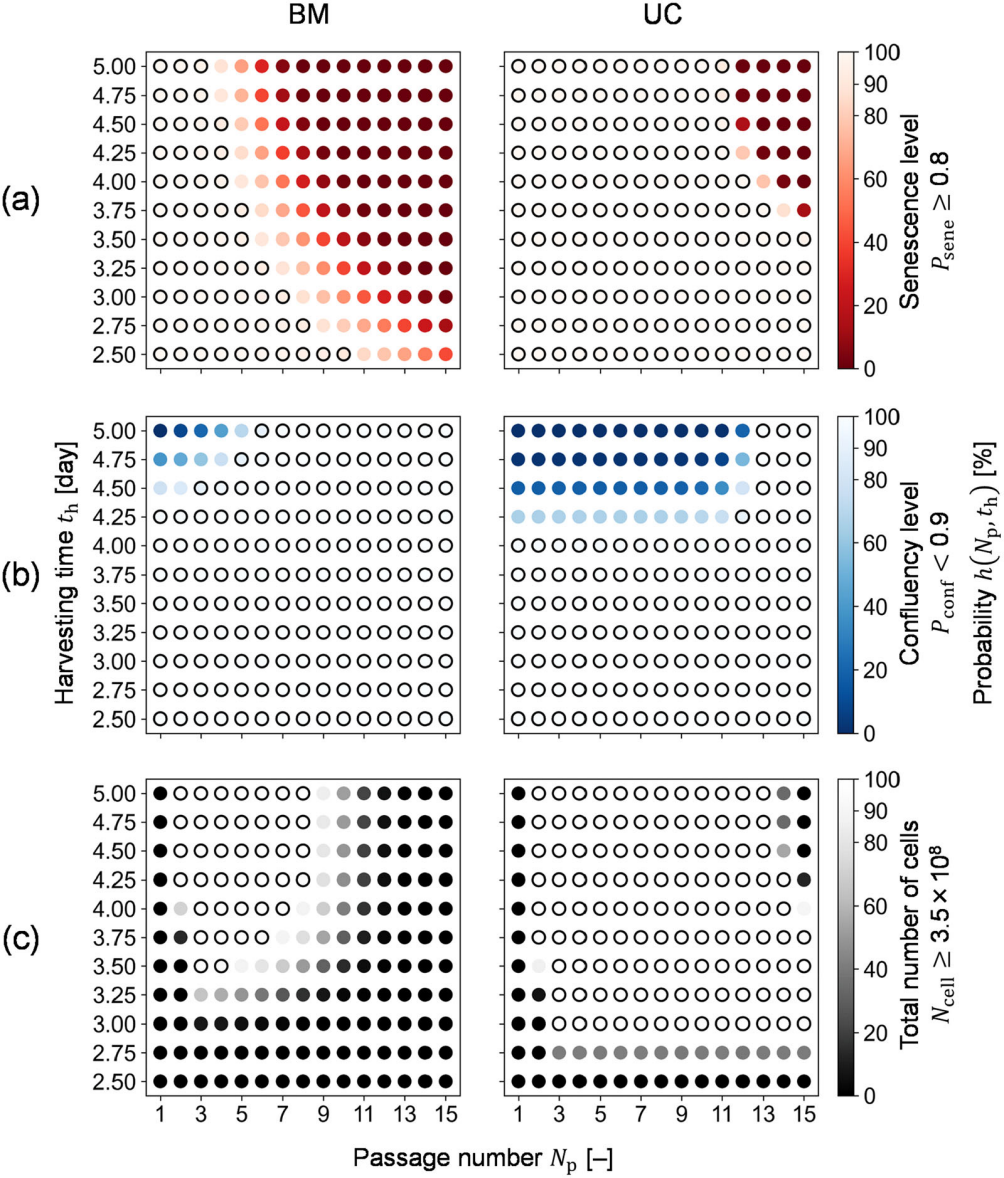
Probability map calculation results. Black solid circles illustrate conditions where the calculated probability is 90% or greater; otherwise, the probability is less than 90%. (a) Result for the senescence level. (b) Result for the confluency level. (c) Result for the total number of cells. BM and UC represent MSCs from bone marrow and umbilical cord, respectively.

Out of the potential 165 conditions, feasible conditions of passage culture were individually specified as DSs for BM‐ and UC‐MSCs. To evaluate the conditions regarding each QI, probability maps for $P_{\text{sene}}$, $P_{\text{conf}}$, and $N_{\text{cell}}$ were obtained with the individual specifications $A_{1}$, $A_{2}$, and $A_{3}$, respectively, instead of the overall specification A (Equation (14) and (15)). In the map for $P_{\text{sene}}$, for example, $t_{h}$ of Day 4 ensured the specification $A_{1}$ with a probability of 90% or greater until passage number 4 and 12 for BM‐ and UC‐MSCs, respectively. These maps identified higher $N_{p}$ values with higher $t_{h}$ as infeasible in terms of senescence (Figure 7a) and lower $N_{p}$ with higher $t_{h}$ as infeasible due to confluency (Figure 7b). The conditions that resulted in too low $P_{\text{sene}}$ (Figure 7a) were identified as infeasible in terms of $N_{\text{cell}}$ (Figure 7c) due to insufficient growth rate. In addition, the map for $N_{\text{cell}}$ indicated that the minimum $N_{p}$ was 2 for both MSCs, whereas the minimum $t_{h}$ values were on Days 3.5 and 3 for BM‐ and UC‐MSCs, respectively (Figure 7c).

**Figure 8:**
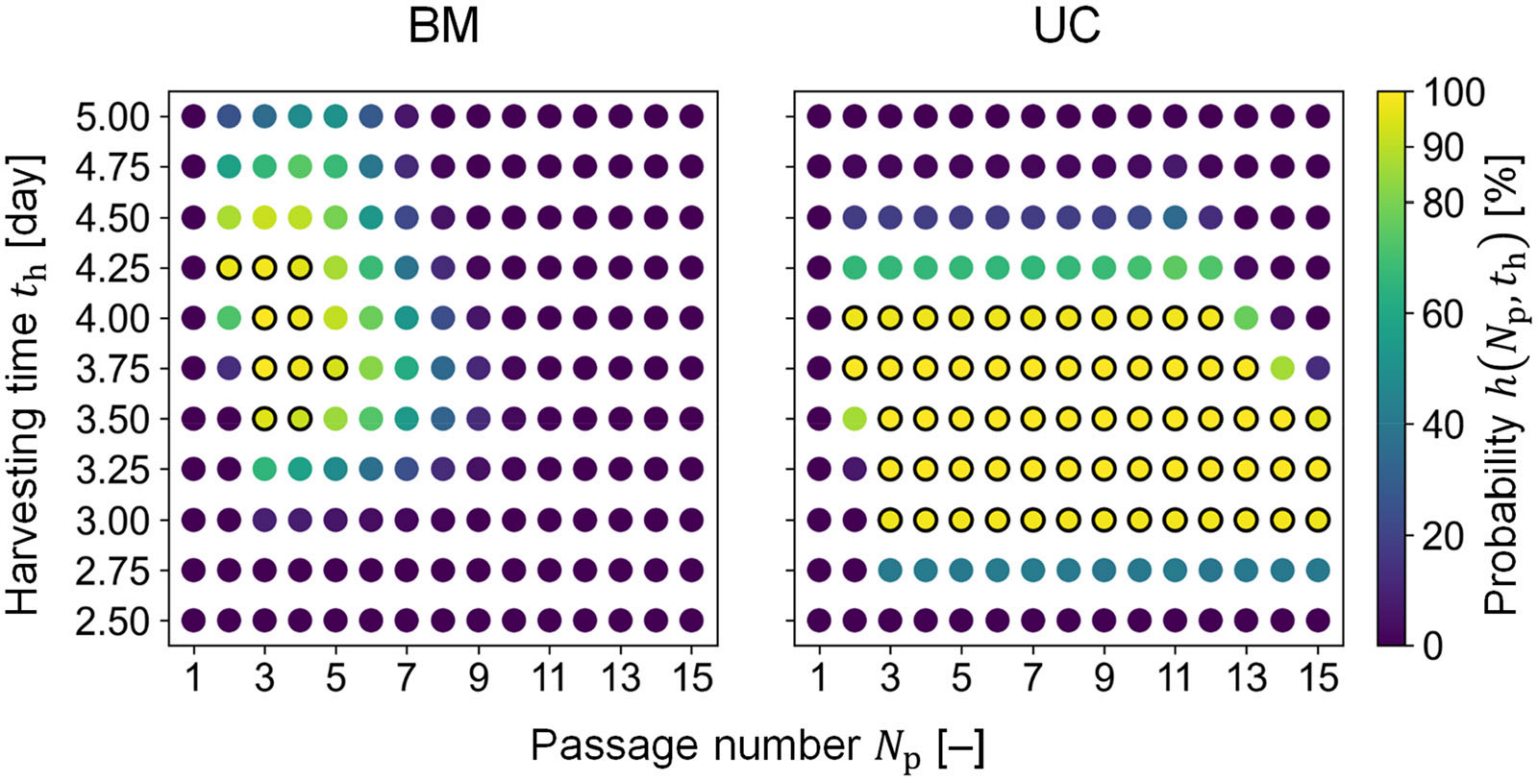
DS determination results. Black solid circles illustrate conditions where the calculated probability is 90% or greater; otherwise, the probability is less than 90%. BM and UC represent MSCs from bone marrow and umbilical cord, respectively.

In addition to the individual probability maps, DSs were determined such that all three specifications were simultaneously ensured with a probability of 90% or greater. A total of 10 and 62 conditions (out of 165) were identified as feasible for BM‐ and UC‐MSCs, respectively (Figure 8). Based on the DSs, feasible ranges of the design variables were identified; for example, when $N_{p}$ was selected as 3, $t_{h}$ with the range of Days 3.5–4.25 and 3–4 were proposed for BM‐ and UC‐MSCs, respectively. Moreover, feasible conditions could be suggested according to user‐specified perspectives such as total time for passage culture; for example, ($N_{p}$, $t_{h}$) of (3, 3.5) and (3, 3) were desirable for BM‐ and UC‐MSCs, with a total time of 10.5 and 9 days, respectively. Other indicators could also be integrated with the DSs to specify preferable conditions. One potential risk associated with the increase in $N_{p}$ is contamination. In this regard, the frequency of operation involved with non‐aseptic manipulations due to the increasing number of passages could be parameterized to further assess the conditions proposed by the DS.

The DS is also influenced by variations of $X_{m}$; specifically, the variation of μ introduced by e (Equation (13)) is lost for a large $t_{h}$ when $X_{h}$ reduces to $X_{m}$, which is constant (see also Equation (2)). To quantify the impacts of the likely variations of $X_{m}$ on the DS, a probability difference, $∆h\left(N_{p},t_{h}\right)$, was calculated by comparing $h\left(N_{p},t_{h}\right)$ to the probability, $h_{2}\left(N_{p},t_{h}\right)$, which incorporated both experimental and cell variabilities (i.e., e and $X_{m}$; Equations (16)–(18)). The variation of $X_{m}$ was represented with a normal distribution applying the specified value (Supporting Information S1: Table S2) as the mean and assuming the standard deviation of 5% based on prior experimental results (Hirono et al. 2024; Jossen et al. 2020) (Equation (19)).

(16) $$∆h\left(N_{p},t_{h}\right)=h_{2}\left(N_{p},t_{h}\right)-h\left(N_{p},t_{h}\right),$$

(17) $$h_{2}\left(N_{p},t_{h}\right)=\frac{1}{M}\sum_{i=1}^{M}I\left(y_{2,i}\in A\right)\times 100\%,$$

(18) $$y_{2}=f\left(N_{p},t_{h},e,X_{m}\right),$$

(19) $$X_{m}\sim N\left({6.41\times 10}^{4},{6.41\times 10}^{4}\times \frac{5}{100}\right),$$

where $y_{2}$ is the model prediction of the QIs (see also Supporting Information S1: Figure S2). The resulting variation in $∆h\left(N_{p},t_{h}\right)$ was less than 2.5% for both BM‐ and UC‐MSCs (Supporting Information S1: Table S3), and the specified variation of $X_{m}$ did not affect the DS in Figure 8. These results suggested that the DS could serve as a basis for industrial MSC passage culture process design.

## 4 Discussion

In this study, we developed a model to predict the growth rate as a function of cPDL for each passage. To our knowledge, this is the first time that the growth rate of each passage is predicted with cPDL, which allows exploring cultivation conditions in each passage as well as passage number. For further model investigations and applications, analyzing the structure and parameters of this model with biological interpretation would be important. By decomposing the model (Equation (4)) into two terms, the maximum specific growth rate and the rest term, this model can be interpreted in accordance with the Monod equations (Monod 1949). Specifically, the growth rate becomes half of the maximum specific growth rate when cPDL reaches $K_{50}$ in the rest term, which is in line with the concept of growth inhibition (Han and Levenspiel 1988). In addition, the first derivative of the remaining term with respect to cPDL is calculated as $-K_{\text{sr}}$, where $\mathrm{cPDL}=K_{50}$, which indicates that $K_{\text{sr}}$ would be relevant to the loss of cell proliferative capacity due to the senescence. This model aims to describe the decreasing trend in the growth rate based on the fundamental phenomena of senescence and shares similar structure/parameters with previous works (Han and Levenspiel 1988; Monod 1949). On the other hand, the model fails to account for an increasing trend in the growth rate during the early passages, especially for UC‐MSCs (see also Figure 4). Further knowledge of senescence could make the model more mechanistic, capturing both the increasing and decreasing trends, and ultimately more robust for passage culture processes.

While the investigated $t_{h}$ to determine the DSs (i.e., Days 2.5–5) differed from the experimental $t_{h}$ (i.e., Day 4), we considered the model outputs reliable. This was because cultivation conditions, more specifically, the effects of nutrient concentrations on the growth rate, during Days 2.5–5 would be regarded as the same as those used for the parameter estimation. Specifically, nutrients (e.g., glucose) in the culture medium were replenished by performing either passage (when $t_{h}\leq$ Day 3) or medium change (when Day 3 $<t_{h}$), complying with the manufacturer's protocol. In this context, such extrapolation of $t_{h}$ would be justified, and thus, the resulting DSs can be useful for the design of industrial MSC passage culture with further validation of the proposed conditions.

In the manufacture of cell‐based products, the concept of cell manufacturability is prominent (Kino‐oka et al. 2019). It is important to predict the quality of cells through the model due to the limitation of proliferative capacity. In this context, the advantage of this model is the ability to predict senescence (i.e., biological parameter) depending on the passage number and harvesting time. Previous passage culture simulations could not specify acceptable ranges of design variables due to a lack of accounting for cPDL, as well as cultivation conditions (Jin et al. 2017; Mehrian et al. 2020). The developed model can suggest acceptable ranges of passage number and harvesting time based on the DS, which would enhance the capability of cell manufacturing processes.

A potential limitation of this study is the large increment in passage model execution. Specifically, the model was executed every harvesting time, which kept cPDL constant during a single cultivation and updated cPDL and μ every harvesting time. This shortcoming can be refined by calculating $P_{\text{sene}}$ using an updated cPDL; namely, the ignored increase in cPDL can be incorporated into the prediction of $P_{\text{sene}}$ and thus in the resulting DS. More numerical investigations and/or higher fidelity models would compensate for this limitation at the cost of more computations.

## 5 Conclusions and Outlook

A novel model for the design of MSC passage culture processes was developed. With the experimental data, the model was applied for the BM‐ and UC‐MSC passage culture process design. The experimentally observed variations in the specific growth rate were incorporated into the stochastic simulation with normal distributions, which predicted variations in the senescence and confluency levels, and in the total number of cells. Finally, feasible conditions of passage numbers and harvesting times were identified, such that the conditions ensured all the quality specifications with 90% or greater.

Future work will include the investigation of unit operations before/after passage culture such as seeding (Scholz et al. 2023) and harvesting (Mawji et al. 2022) toward an MSC manufacturing process design. More experimental and numerical investigations could also correlate the passage model parameters with cell‐source information (Mehrian et al. 2020) such as donor age (Zaim et al. 2012) and gender (Fossett et al. 2012), which would be relevant to industrial MSC manufacturing.

## Nomenclature

### Symbols

A
: (–) set of quality specifications
$A_{1}$
: (–) quality specification of senescence level
$A_{2}$
: (–) quality specification of confluency level
$A_{3}$
: (–) quality specification of the total number of cells
e
: (h^–1^) random variable representing variability
f
: (–) developed model
h
: (%) calculated probability
I
: (–) indicator function
$K_{50}$
: (–) passage model constant
$K_{\text{sr}}$
: (–) senescence rate
$L^{n}$
: (–) every combination of design variable
m
: (–) number of measurements
M
: (–) number of model iterations
n
: (–) number of samples
$n_{\text{flask}}$
: (–) number of working flasks
$n_{\text{flask}}^{\max}$
: (–) maximum number of available flasks
$n_{\text{flask}}^{\text{next}}$
: (–) number of flasks for next cultivation
$N_{\text{cell}}$
: (cells) total number of cells
$N_{p}$
: (–) passage number
N(a,b)
: (–) normal distribution with a mean of a and standard deviation of b
$P_{\text{conf}}$
: (–) confluency level
$P_{\text{sene}}$
: (–) senescence level
S
: (cm^2^) surface area of the flask
$\bar{\mathrm{SD}}$
: (h^–1^) mean of sample standard deviations
t
: (h) cultivation time
$t_{h}$
: (day) harvesting time
X
: (cells cm^–2^) cell density
$X_{h}$
: (cells cm^–2^) harvesting density
$X_{m}$
: (cells cm^–2^) maximum cell density
$X_{s}$
: (cells cm^–2^) seeding density
y
: (–) stochastic simulation results

### 1 Greek Letters

μ
: (h^–1^) specific growth rate
$\mu_{m}$
: (h^–1^) maximum specific growth rate
$\mu^{\text{exp}}$
: (h^–1^) specific growth rate obtained from experimental data
$\bar{\mu^{\text{exp}}}$
: (h^–1^) mean of specific growth rate obtained from experimental data
π
: (%) minimum acceptable risk

## Author Contributions

**Keita Hirono:** conceptualization, data curation, formal analysis, investigation, methodology, software, validation, visualization, writing – original draft, writing – review and editing. **Yusuke Hayashi:** conceptualization, methodology, validation, visualization, writing – original draft, writing – review and editing. **Yuuki Ogawa:** conceptualization, data curation, formal analysis, investigation, methodology, validation, visualization, writing – original draft, writing – review and editing. **Masahiro Kino‐oka:** conceptualization, funding acquisition, project administration, resources, supervision. **Hirokazu Sugiyama:** conceptualization, funding acquisition, methodology, project administration, resources, supervision, visualization, writing – review and editing.

## Conflicts of Interest

The authors declare no conflicts of interest.

## Supporting information

Hirono_etal_SI_Revision.

## Data Availability

All data supporting the findings of this study are available within the article and its Supplementary Information. The raw data of the experiments are also available from the corresponding authors upon reasonable request.

## References

[bit29001-bib-0001] Bonab, M. M. , K. Alimoghaddam , F. Talebian , S. H. Ghaffari , A. Ghavamzadeh , and B. Nikbin . 2006. “Aging of Mesenchymal Stem Cell in Vitro.” BMC Cell Biology 7: 14. 16529651 10.1186/1471-2121-7-14 PMC1435883

[bit29001-bib-0002] Bruder, S. P. , N. Jaiswal , and S. E. Haynesworth . 1997. “Growth Kinetics, Self‐Renewal, and the Osteogenic Potential of Purified Human Mesenchymal Stem Cells During Extensive Subcultivation and Following Cryopreservation.” Journal of Cellular Biochemistry 64, no. 2: 278–294. 9027588 10.1002/(sici)1097-4644(199702)64:2<278::aid-jcb11>3.0.co;2-f

[bit29001-bib-0003] Destro, F. , and M. Barolo . 2022. “A Review on the Modernization of Pharmaceutical Development and Manufacturing ‐ Trends, Perspectives, and the Role of Mathematical Modeling.” International Journal of Pharmaceutics 620: 121715. 35367580 10.1016/j.ijpharm.2022.121715

[bit29001-bib-0004] Digirolamo, C. M. , D. Stokes , D. Colter , D. G. Phinney , R. Class , and D. J. Prockop . 1999. “Propagation and Senescence of Human Marrow Stromal Cells in Culture: A Simple Colony‐Forming Assay Identifies Samples With the Greatest Potential to Propagate and Differentiate.” British Journal of Haematology 107, no. 2: 275–281. 10583212 10.1046/j.1365-2141.1999.01715.x

[bit29001-bib-0005] Fossett, E. , W. S. Khan , U. G. Longo , and P. J. Smitham . 2012. “Effect of Age and Gender on Cell Proliferation and Cell Surface Characterization of Synovial Fat Pad Derived Mesenchymal Stem Cells.” Journal of Orthopaedic Research 30, no. 7: 1013–1018. 22228598 10.1002/jor.22057

[bit29001-bib-0006] García‐Muñoz, S. , C. V. Luciani , S. Vaidyaraman , and K. D. Seibert . 2015. “Definition of Design Spaces Using Mechanistic Models and Geometric Projections of Probability Maps.” Organic Process Research & Development 19, no. 8: 1012–1023.

[bit29001-bib-0007] Han, K. , and O. Levenspiel . 1988. “Extended Monod Kinetics for Substrate, Product, and Cell Inhibition.” Biotechnology and Bioengineering 32, no. 4: 430–447. 18587739 10.1002/bit.260320404

[bit29001-bib-0008] Hayflick, L. 1965. “The Limited in Vitro Lifetime of Human Diploid Cell Strains.” Experimental Cell Research 37, no. 3: 614–636. 14315085 10.1016/0014-4827(65)90211-9

[bit29001-bib-0009] Heathman, T. R. , A. W. Nienow , M. J. McCall , K. Coopman , B. Kara , and C. J. Hewitt . 2015. “The Translation of Cell‐Based Therapies: Clinical Landscape and Manufacturing Challenges.” Regenerative Medicine 10, no. 1: 49–64. 25562352 10.2217/rme.14.73

[bit29001-bib-0010] Hirono, K. , Y. Hayashi , I. A. Udugama , et al. 2024. “Image‐Based Hybrid Model Incorporating Initial Spatial Distribution for Mesenchymal Stem Cell Cultivation Process Design.” AIChE Journal 70, no. 7: e18452.

[bit29001-bib-0011] Hirono, K. , I. A. Udugama , Y. Hayashi , M. Kino‐oka , and H. Sugiyama . 2022. “A Dynamic and Probabilistic Design Space Determination Method for Mesenchymal Stem Cell Cultivation Processes.” Industrial & Engineering Chemistry Research 61, no. 20: 7009–7019.

[bit29001-bib-0012] ICH . (2009, August 1). *Q8(R2) Pharmaceutical Development*. https://vnras.com/wp-content/uploads/2017/05/Q8R2_PHARMACEUTICAL-DEVELOPMENT.pdf.

[bit29001-bib-0013] Jin, W. , C. J. Penington , S. W. McCue , and M. J. Simpson . 2017. “A Computational Modelling Framework to Quantify the Effects of Passaging Cell Lines.” PloS One 12, no. 7: e0181941. 28750084 10.1371/journal.pone.0181941 PMC5531485

[bit29001-bib-0014] Jossen, V. , F. Muoio , S. Panella , Y. Harder , T. Tallone , and R. Eibl . 2020. “An Approach Towards a GMP Compliant In‐Vitro Expansion of Human Adipose Stem Cells for Autologous Therapies.” Bioengineering 7, no. 3: 77–99. 32698363 10.3390/bioengineering7030077 PMC7552624

[bit29001-bib-0015] Kern, S. , H. Eichler , J. Stoeve , H. Klüter , and K. Bieback . 2006. “Comparative Analysis of Mesenchymal Stem Cells From Bone Marrow, Umbilical Cord Blood, or Adipose Tissue.” STEM CELLS 24, no. 5: 1294–1301. 16410387 10.1634/stemcells.2005-0342

[bit29001-bib-0016] Kino‐oka, M. , M. Mizutani , and N. Medcalf . 2019. “Cell Manufacturability.” Cell & Gene Therapy Insights 5, no. 10: 1347–1359.

[bit29001-bib-0017] Li, X.‐Y. , J. Ding , Z.‐H. Zheng , X.‐Y. Li , Z.‐B. Wu , and P. Zhu . 2012. “Long‐Term Culture in Vitro Impairs the Immunosuppressive Activity of Mesenchymal Stem Cells on T Cells.” Molecular Medicine Reports 6, no. 5: 1183–1189. 22923041 10.3892/mmr.2012.1039

[bit29001-bib-0018] Lipsitz, Y. Y. , N. E. Timmins , and P. W. Zandstra . 2016. “Quality Cell Therapy Manufacturing by Design.” Nature Biotechnology 34, no. 4: 393–400. 10.1038/nbt.3525 27054995

[bit29001-bib-0019] Mawji, I. , E. L. Roberts , T. Dang , B. Abraham , and M. S. Kallos . 2022. “Challenges and Opportunities In Downstream Separation Processes for Mesenchymal Stromal Cells Cultured in Microcarrier‐Based Stirred Suspension Bioreactors.” Biotechnology and Bioengineering 119, no. 11: 3062–3078. 35962467 10.1002/bit.28210

[bit29001-bib-0020] Mehrian, M. , T. Lambrechts , M. Marechal , F. P. Luyten , I. Papantoniou , and L. Geris . 2020. “Predicting in Vitro Human Mesenchymal Stromal Cell Expansion Based on Individual Donor Characteristics Using Machine Learning.” Cytotherapy 22, no. 2: 82–90. 31987754 10.1016/j.jcyt.2019.12.006

[bit29001-bib-0021] Mets, T. , and G. Verdonk . 1981. “In Vitro Aging of Human Bone Marrow Derived Stromal Cells.” Mechanisms of Ageing and Development 16, no. 1: 81–89. 7253722 10.1016/0047-6374(81)90035-x

[bit29001-bib-0022] Monod, J. 1949. “The Growth OF Bacterial Cultures.” Annual Review of Microbiology 3, no. 1: 371–394.

[bit29001-bib-0023] Pittenger, M. F. , A. M. Mackay , S. C. Beck , et al. 1999. “Multilineage Potential of Adult Human Mesenchymal Stem Cells.” Science 284, no. 5411: 143–147. 10102814 10.1126/science.284.5411.143

[bit29001-bib-0024] Scholz, B. X. , Y. Hayashi , I. A. Udugama , M. Kino‐oka , and H. Sugiyama . 2023. “A CFD Model‐Based Design of Seeding Processes for Two‐Dimensional Mesenchymal Stem Cell Cultivation.” Computers & Chemical Engineering 171: 108157.

[bit29001-bib-0025] Wiese, D. M. , C. C. Ruttan , C. A. Wood , B. N. Ford , and L. R. Braid . 2019. “Accumulating Transcriptome Drift Precedes Cell Aging in Human Umbilical Cord‐Derived Mesenchymal Stromal Cells Serially Cultured to Replicative Senescence.” Stem Cells Translational Medicine 8, no. 9: 945–958. 30924318 10.1002/sctm.18-0246 PMC6708062

[bit29001-bib-0026] Zaim, M. , S. Karaman , G. Cetin , and S. Isik . 2012. “Donor Age and Long‐Term Culture Affect Differentiation and Proliferation of Human Bone Marrow Mesenchymal Stem Cells.” Annals of Hematology 91, no. 8: 1175–1186. 22395436 10.1007/s00277-012-1438-x

