# Supplementary material for: A Mathematical Model for Determining Probabilistic Design Space in Mesenchymal Stem Cell Passage Culture
Source: Biotechnol Bioeng. 2025 Apr 25;122(8):2009–20. doi: 10.1002/bit.29001 (PMC12235243; doi:10.1002/bit.29001)
Supplement: Supplementary file 1 — Hirono_etal_SI_Revision. [file BIT-122-2009-s001.docx]

**Supporting Information**

**A Mathematical Model for Determining Probabilistic Design Space in Mesenchymal Stem Cell Passage Culture**

Keita Hirono^1^, Yusuke Hayashi^1^, Yuuki Ogawa^2^, Masahiro Kino-oka^2^, and Hirokazu Sugiyama^1,*^

^1^*Department of Chemical System Engineering, The University of Tokyo, 7-3-1, Hongo, Bunkyo-ku, Tokyo 113-8656, Japan*

^2^*Department of Biotechnology, Graduate School of Engineering, Osaka University, 1-2, Yamadaoka, Suita-shi, Osaka 565-0871, Japan*

^*^Corresponding author

sugiyama@chemsys.t.u-tokyo.ac.jp

TEL & FAX: +81-3-5841-7227

# FIGURE S1 | Parameter estimation and model validation results using different sets of experimental samples. (a) Estimation with samples 2 & 3 and validation with sample 1, (b) estimation with samples 3 & 1 and validation with sample 2, color code: white (sample 1), gray (sample 2), and black (sample 3). Red dashed lines represent the model fit. Red diamond plots show the model prediction. NRMSE is the normalized root mean square error. BM and UC represent MSCs from bone marrow and umbilical cord, respectively.

# FIGURE S2 | Stochastic simulation with the harvesting time of Day 5 without/with the distribution of the maximum cell density, $\boldsymbol{X}_{\mathbf{m}}$. (a) Results for the harvesting density and the cumulative population doubling level. (b) Results for the QIs of the senescence level, the confluency level, and the total number of cells, where the “Without” results are also presented in Figure 7. BM and UC represent MSCs from bone marrow and umbilical cord, respectively.

# TABLE S1 | Estimated parameter values using different sets of experimental samples. Sample ID shows samples used to estimate the parameters. BM and UC represent MSCs from bone marrow and umbilical cord, respectively.

| Cell source | Sample ID | Maximum specific growth rate $\mu_{m}$ [10^–2^ h^–1^] | Senescence rate $K_{\mathrm{sr}}$ [–] | Senescence model constant $K_{50}$ [–] |
| --- | --- | --- | --- | --- |
| BM | 1 & 2 | 2.82 | 0.110 | 28.3 |
|  | 2 & 3 | 2.75 | 0.126 | 29.8 |
|  | 3 & 1 | 2.82 | 0.126 | 29.5 |
| UC | 1 & 2 | 3.27 | 0.331 | 49.0 |
|  | 2 & 3 | 3.27 | 0.262 | 49.1 |
|  | 3 & 1 | 3.34 | 0.404 | 49.9 |

# TABLE S2 | Specified maximum cell density values. BM and UC represent MSCs from bone marrow and umbilical cord; $\boldsymbol{N}_{\mathbf{p}}$ is the passage number, and n/a is “no available data.”

| $N_{p}$ | Cell density [10^4^ cells cm^–2^] | | | | | |
| --- | --- | --- | --- | --- | --- | --- |
|  | BM samples | | | UC samples | | |
|  | 1 | 2 | 3 | 1 | 2 | 3 |
| 1 | 3.10 | 2.97 | 2.99 | 2.91 | 2.86 | 3.57 |
| 2 | 3.24 | 3.99 | 3.76 | 5.14 | 6.15 | 5.10 |
| 3 | 3.43 | 3.20 | 3.71 | 4.19 | 3.79 | 4.78 |
| 4 | 3.45 | 2.65 | 3.82 | 3.88 | 3.79 | 3.84 |
| 5 | 3.33 | 2.30 | 2.40 | 3.57 | 4.06 | 2.32 |
| 6 | 2.22 | 2.28 | 2.58 | 3.48 | 3.21 | 4.18 |
| 7 | 2.02 | 2.55 | 2.40 | 5.57 | 5.47 | 6.04 |
| 8 | 2.53 | 2.06 | 2.06 | 7.28 | 5.33 | 5.05 |
| 9 | 1.71 | 1.57 | 1.80 | 3.48 | 2.95 | 4.47 |
| 10 | 1.65 | 2.10 | 2.24 | 6.35 | 4.92 | 4.69 |
| 11 | 1.53 | 1.68 | 1.92 | 5.46 | 5.38 | 4.87 |
| 12 | 1.16 | 1.38 | 1.62 | 7.73 | 3.52 | 5.09 |
| 13 | 0.915 | 1.16 | 1.26 | 6.00 | 4.11 | 3.97 |
| 14 | 1.09 | 1.16 | 1.35 | 4.47 | 2.62 | 2.24 |
| 15 | 1.06 | 1.58 | 1.68 | 3.04 | 2.07 | 2.37 |
| 16 | 0.771 | 0.503 | 0.491 | 1.30 | 2.02 | 1.10 |
| 17 | 0.556 | 0.852 | 1.17 | 1.11 | 1.32 | 0.894 |
| 18 | 0.507 | 0.753 | 0.524 | n/a | n/a | n/a |
| 19 | 0.471 | 0.681 | 0.471 | n/a | n/a | n/a |
| 20 | 0.523 | 0.573 | 0.483 | n/a | n/a | n/a |
| 21 | 0.423 | 0.633 | 0.684 | n/a | n/a | n/a |
| 22 | 0.780 | 0.423 | 0.617 | n/a | n/a | n/a |
| Maximum value of the experimental data | 3.99 | | | 7.73 | | |
| Literature value | 6.41  (Hirono et al., 2024) | | | n/a | | |
| Maximum cell density in this work | 6.41 | | | 7.73 | | |

# TABLE S3 | Results of the calculated probability difference, $\boldsymbol{\Delta h}\left( \boldsymbol{N}_{\mathbf{p}}\boldsymbol{,}\boldsymbol{t}_{\mathbf{h}} \right)$, where $\boldsymbol{h}_{\mathbf{2}}\left( \boldsymbol{N}_{\mathbf{p}}\boldsymbol{,}\boldsymbol{t}_{\mathbf{h}} \right)$ is the probability which incorporated both experimental and cell variabilities. BM and UC represent MSCs from bone marrow and umbilical cord, respectively.

| Passage number  $N_{p}$  [–] | Harvesting time $t_{h}$ | | | | | |
| --- | --- | --- | --- | --- | --- | --- |
|  | Day 2.5 | | Day 3 | | Day 3.5 | |
|  | $h_{2}\left( N_{p},t_{h} \right)$ [%] | $\Delta h\left( N_{p},t_{h} \right)$ [%] | $h_{2}\left( N_{p},t_{h} \right)$ [%] | $\Delta h\left( N_{p},t_{h} \right)$ [%] | $h_{2}\left( N_{p},t_{h} \right)$ [%] | $\Delta h\left( N_{p},t_{h} \right)$ [%] |
| BM |  |  |  |  |  |  |
| 1 | 0 | 0 | 0 | 0 | 0 | 0 |
| 2 | 0 | 0 | 0 | 0 | 0.11 | -0.01 |
| 3 | 0 | 0 | 9.45 | 0.51 | 93.67 | 0.18 |
| 4 | 0 | 0 | 8.44 | 0.17 | 90.40 | -0.13 |
| 5 | 0 | 0 | 5.59 | 0.21 | 82.98 | -0.59 |
| 6 | 0 | 0 | 3.41 | -0.09 | 71.08 | -0.87 |
| 7 | 0 | 0 | 2.10 | -0.03 | 53.23 | 0.08 |
| 8 | 0 | 0 | 0.94 | 0.01 | 30.65 | -0.53 |
| 9 | 0 | 0 | 0.42 | 0.11 | 11.98 | 0.05 |
| 10 | 0 | 0 | 0.10 | 0 | 2.45 | -0.09 |
| 11 | 0 | 0 | 0.01 | -0.02 | 0.37 | -0.03 |
| 12 | 0 | 0 | 0 | 0 | 0 | -0.03 |
| 13 | 0 | 0 | 0 | 0 | 0 | 0 |
| 14 | 0 | 0 | 0 | 0 | 0 | 0 |
| 15 | 0 | 0 | 0 | 0 | 0 | 0 |
| UC |  |  |  |  |  |  |
| 1 | 0 | 0 | 0 | 0 | 0 | 0 |
| 2 | 0 | 0 | 0 | 0 | 85.81 | -0.87 |
| 3 | 0.02 | 0.02 | 98.28 | 0.32 | 99.99 | 0 |
| 4 | 0.03 | -0.02 | 98.04 | -0.08 | 99.98 | -0.01 |
| 5 | 0.03 | -0.04 | 98.21 | 0.12 | 99.99 | -0.01 |
| 6 | 0.01 | -0.03 | 98.05 | -0.34 | 100 | 0.02 |
| 7 | 0.02 | -0.04 | 98.10 | -0.02 | 99.96 | -0.03 |
| 8 | 0.03 | -0.06 | 98.06 | -0.01 | 100 | 0.02 |
| 9 | 0.06 | 0.02 | 97.89 | -0.36 | 99.97 | -0.02 |
| 10 | 0.04 | 0.01 | 98.07 | -0.21 | 100 | 0.03 |
| 11 | 0.03 | 0.01 | 98.05 | -0.22 | 100 | 0.01 |
| 12 | 0.05 | 0.03 | 98.17 | -0.02 | 99.98 | 0.01 |
| 13 | 0.04 | 0.02 | 98.01 | -0.13 | 99.96 | 0.02 |
| 14 | 0.07 | 0.05 | 98.07 | -0.01 | 99.78 | 0.04 |
| 15 | 0.03 | 0 | 98.03 | 0.59 | 96.25 | 0.03 |

| Passage number  $N_{p}$  [–] | Harvesting time $t_{h}$ | | | | | |
| --- | --- | --- | --- | --- | --- | --- |
|  | Day 4 | | Day 4.5 | | Day 5 | |
|  | $h_{2}\left( N_{p},t_{h} \right)$ [%] | $\Delta h\left( N_{p},t_{h} \right)$ [%] | $h_{2}\left( N_{p},t_{h} \right)$ [%] | $\Delta h\left( N_{p},t_{h} \right)$ [%] | $h_{2}\left( N_{p},t_{h} \right)$ [%] | $\Delta h\left( N_{p},t_{h} \right)$ [%] |
| BM |  |  |  |  |  |  |
| 1 | 0 | 0 | 0 | 0 | 0 | 0 |
| 2 | 70.17 | -0.69 | 85.63 | -0.30 | 26.01 | 0.79 |
| 3 | 98.06 | -0.19 | 88.26 | -1.59 | 34.46 | 0.14 |
| 4 | 95.20 | -0.95 | 87.74 | -0.81 | 46.07 | -0.78 |
| 5 | 89.37 | 0.15 | 77.85 | -0.31 | 48.93 | -1.10 |
| 6 | 75.56 | -0.56 | 52.96 | 0.44 | 27.39 | -0.11 |
| 7 | 51.31 | -0.13 | 21.73 | -0.47 | 6.25 | 0.13 |
| 8 | 24.02 | 0.33 | 4.92 | -0.05 | 0.73 | 0.12 |
| 9 | 5.84 | 0.11 | 0.48 | 0 | 0 | 0 |
| 10 | 0.53 | -0.21 | 0.03 | 0.02 | 0 | 0 |
| 11 | 0.02 | 0 | 0 | 0 | 0 | 0 |
| 12 | 0 | 0 | 0 | 0 | 0 | 0 |
| 13 | 0 | 0 | 0 | 0 | 0 | 0 |
| 14 | 0 | 0 | 0 | 0 | 0 | 0 |
| 15 | 0 | 0 | 0 | 0 | 0 | 0 |
| UC |  |  |  |  |  |  |
| 1 | 0 | 0 | 0 | 0 | 0 | 0 |
| 2 | 96.42 | -1.08 | 20.16 | 2.24 | 0.14 | 0.05 |
| 3 | 96.59 | -1.04 | 19.24 | 0.73 | 0.14 | 0.07 |
| 4 | 96.66 | -0.98 | 18.69 | 0.27 | 0.15 | 0.07 |
| 5 | 96.73 | -0.89 | 18.98 | 1.11 | 0.22 | 0.07 |
| 6 | 97.03 | -0.54 | 19.09 | 1.30 | 0.18 | 0.11 |
| 7 | 96.63 | -0.88 | 19.04 | 1.14 | 0.07 | -0.06 |
| 8 | 96.98 | -0.91 | 19.84 | 1.40 | 0.15 | 0.09 |
| 9 | 96.86 | -1.05 | 20.79 | 1.96 | 0.16 | 0.02 |
| 10 | 96.99 | -0.70 | 23.15 | 0.70 | 0.26 | 0.04 |
| 11 | 97.25 | -1.04 | 35.22 | 0.91 | 0.98 | 0.20 |
| 12 | 97.83 | -0.76 | 13.49 | 0.43 | 0.37 | 0.03 |
| 13 | 77.09 | 0.13 | 0 | 0 | 0 | 0 |
| 14 | 3.72 | 0.24 | 0 | 0 | 0 | 0 |
| 15 | 0 | 0 | 0 | 0 | 0 | 0 |

# Reference

Hirono, K., Hayashi, Y., A. Udugama, I., Takemoto, Y., Kato, R., Kino-oka, M., & Sugiyama, H. (2024). Image-based hybrid model incorporating initial spatial distribution for mesenchymal stem cell cultivation process design. *AIChE Journal*, *70*(7), e18452.
